# Dynamic inflammatory markers as predictors of 90-day outcomes in spontaneous intracerebral hemorrhage

**DOI:** 10.3389/fneur.2026.1787030

**Published:** 2026-06-18

**Authors:** Huiying Huang, Weijun Wang, Qing Ma, Kun Cao

**Affiliations:** 1 Department of Neurology, People's Hospital of Leshan, Leshan, Sichuan, China; 2 Department of Neurology, North Sichuan Medical College, Nanchong, Sichuan, China; 3 Department of Neurology, People's Hospital of Daying County, Suining, Sichuan, China

**Keywords:** composite inflammatory biomarkers, dynamic changes, functional outcome, prognostic assessment, spontaneous intracerebral hemorrhage

## Abstract

**Objective:**

To investigate the time-dependent characteristics of composite inflammatory biomarkers in patients with spontaneous intracerebral hemorrhage (ICH), and to evaluate the associations of their dynamic changes with 90-day functional outcomes and mortality.

**Methods:**

This single-center retrospective study consecutively enrolled 230 patients with spontaneous ICH admitted between 2021 and 2024. Peripheral blood samples were collected within 24 h after admission (T1) and on day 7 after onset (T2). The neutrophil-to-lymphocyte ratio (NLR), systemic immune-inflammation index (SII), systemic inflammation response index (SIRI), and inflammation prognostic index (IPI) were calculated at both time points. Dynamic changes from T1 to T2 were assessed using fold change (FC = T2/T1) and relative percentage change, defined as c1–2 = (T2 − T1) / T1 × 100%, equivalent to (FC − 1) × 100%. Functional outcomes at 90 days were assessed using the modified Rankin Scale, with favorable outcome defined as mRS 0–2 and unfavorable outcome defined as mRS 3–6. Mortality was defined as mRS 6. Logistic regression and receiver operating characteristic curve analyses were performed.

**Results:**

Levels of NLR, SII, SIRI, and IPI at T2 were higher in patients with unfavorable outcomes and in non-survivors than in patients with favorable outcomes and survivors, respectively. Dynamic changes from T1 to T2, assessed using FC and c1–2, were also more pronounced in patients with unfavorable outcomes and in non-survivors. Multivariate analysis showed that T2 levels and c1–2 indicators were significantly associated with 90-day unfavorable functional outcomes. For mortality, T2 indices showed more consistent independent associations than T1 indices, and most dynamic change indicators remained significant after adjustment. ROC analysis indicated that T2 indices and dynamic change indicators generally had better predictive performance than T1 indices.

**Conclusion:**

Composite inflammatory biomarkers in patients with spontaneous ICH exhibit a time-dependent pattern in prognostic evaluation. Compared with baseline measurements alone, day-7 inflammatory levels and their dynamic changes from T1 to T2 show more stable associations with 90-day unfavorable functional outcomes and mortality, and may provide useful complementary information for clinical risk stratification.

## Introduction

Spontaneous intracerebral hemorrhage (ICH) refers to non-traumatic bleeding within the brain parenchyma, which may extend into the ventricular system. After excluding secondary causes such as large-vessel lesions, tumors, infections/inflammatory conditions, or hemodynamic abnormalities, it is generally considered to be associated with cerebral small vessel disease (1). ICH is the second most common type of stroke, accounting for approximately 28.8% of all strokes. The global incidence of ICH ranges from 27 to 30 cases per 100,000 person-years, characterized by rapid onset, severe clinical course, and high mortality and disability rates (2, 3). Previous studies have identified major risk factors for ICH, including elevated systolic blood pressure, increased body mass index, air pollution, diabetes, and smoking (4). Despite advances in blood pressure management, hemostatic therapy, and surgical interventions, there is still a lack of specific treatments that significantly improve long-term prognosis. A considerable proportion of patients continue to experience varying degrees of neurological deficits, and the mortality and disability rates remain high over time (5, 6). Therefore, identifying simple, reproducible, and predictive early prognostic markers is of great importance for optimizing clinical management and risk stratification in ICH patients.

Increasing evidence indicates that inflammatory responses are involved in the development and progression of secondary brain injury following ICH (7). After hemorrhage, the hematoma and its degradation products rapidly activate innate immune responses, leading to the recruitment of peripheral immune cells and amplification of both local and systemic inflammatory responses (8). Traditional inflammatory markers in peripheral blood, including neutrophils, lymphocytes, monocytes, and C-reactive protein (CRP), have been shown to be closely associated with perihematomal edema formation, exacerbation of secondary injury, and unfavorable outcomes after ICH (9–11).

On this basis, a series of composite inflammatory indices derived from routine hematological parameters have been proposed in recent years. The neutrophil-to-lymphocyte ratio (NLR) reflects the balance between neutrophil-mediated innate immune responses and lymphocyte-mediated adaptive immune responses, and its elevation generally indicates enhanced inflammatory activation and immune imbalance (12). The systemic immune-inflammation index (SII), integrating platelet, neutrophil, and lymphocyte counts, reflects both inflammatory responses and thrombo-inflammatory interactions (13). The systemic inflammation response index (SIRI), composed of neutrophils, monocytes, and lymphocytes, is considered to represent the balance between myeloid inflammatory responses and adaptive immune status (14). The inflammation prognostic index (IPI), incorporating CRP, NLR, and albumin, is thought to comprehensively reflect inflammatory activity, immune status, and systemic nutritional condition, and may therefore provide a more comprehensive assessment of the dynamic inflammatory burden following ICH compared with single markers (15).

Previous studies have shown that NLR is associated with unfavorable functional outcomes and 90-day mortality in patients with ICH, and its prognostic value has been further supported by systematic reviews and meta-analyses (9, 16). SII has also been reported as an independent predictor of unfavorable discharge outcomes or 90-day functional outcomes in spontaneous ICH (17). Similarly, SIRI has been shown to be significantly associated with unfavorable outcomes and mortality and may serve as an independent predictor (18, 19). In contrast, evidence regarding IPI in spontaneous ICH remains limited. However, in patients with acute ischemic stroke undergoing intravenous thrombolysis, IPI has been reported to be associated with unfavorable 90-day outcomes (20), suggesting that these composite indices may have potential cross-stroke applicability and providing a theoretical basis for their use in ICH. Nevertheless, studies specifically focusing on patients with spontaneous ICH remain relatively limited.

Most existing studies have focused on inflammatory marker levels at a single time point, while overlooking the dynamic changes in inflammatory responses during disease progression. In fact, post-ICH inflammation is not a static process; its intensity and duration may vary substantially depending on disease progression, complications, and treatment responses. A single baseline measurement may therefore be insufficient to fully capture this complex and dynamic immunoinflammatory process. In contrast, temporal changes in inflammatory markers during the early hospitalization period may provide more clinically meaningful prognostic information. To date, systematic investigations into the relationship between time-dependent inflammatory burden and long-term functional outcomes and mortality in ICH remain limited.

In light of this, the present study evaluated the levels of NLR, SII, SIRI, and IPI at early admission (T1) and on day 7 after onset (T2), as well as their dynamic changes, based on routine peripheral blood tests. We aimed to explore the associations between these dynamic inflammatory indices and 90-day functional outcomes and mortality, and to assess their potential value in prognostic stratification in patients with ICH.

## Materials and methods

### Study design and participants

This study was a single-center retrospective cohort study that consecutively enrolled patients with spontaneous intracerebral hemorrhage (ICH) who were hospitalized at Leshan People’s Hospital between January 2021 and December 2024. The diagnosis of spontaneous ICH was based on the *Chinese Guidelines for the Diagnosis and Treatment of Intracerebral Hemorrhage* and relevant international guidelines, and was confirmed by head computed tomography (CT) or magnetic resonance imaging (MRI).

The inclusion criteria were as follows: (1) age ≥18 years; (2) diagnosis of spontaneous ICH; (3) premorbid modified Rankin Scale (mRS) score ≤2; (4) availability of complete baseline clinical data, imaging data, and 90-day follow-up outcomes; (5) completion of hematological testing within 24 h after admission (T1) and on day 7 after onset (T2). The exclusion criteria were as follows: (1) secondary ICH, including trauma, arteriovenous malformation, aneurysmal rupture, or tumor-related hemorrhage; (2) presence of definite acute or chronic infection at admission; (3) severe hepatic or renal dysfunction; (4) recent major surgery or severe trauma prior to onset; (5) long-term use of glucocorticoids or immunosuppressive agents; (6) missing key baseline clinical variables, imaging data, 90-day follow-up data, or day 7 hematological results.

**Figure 1:**
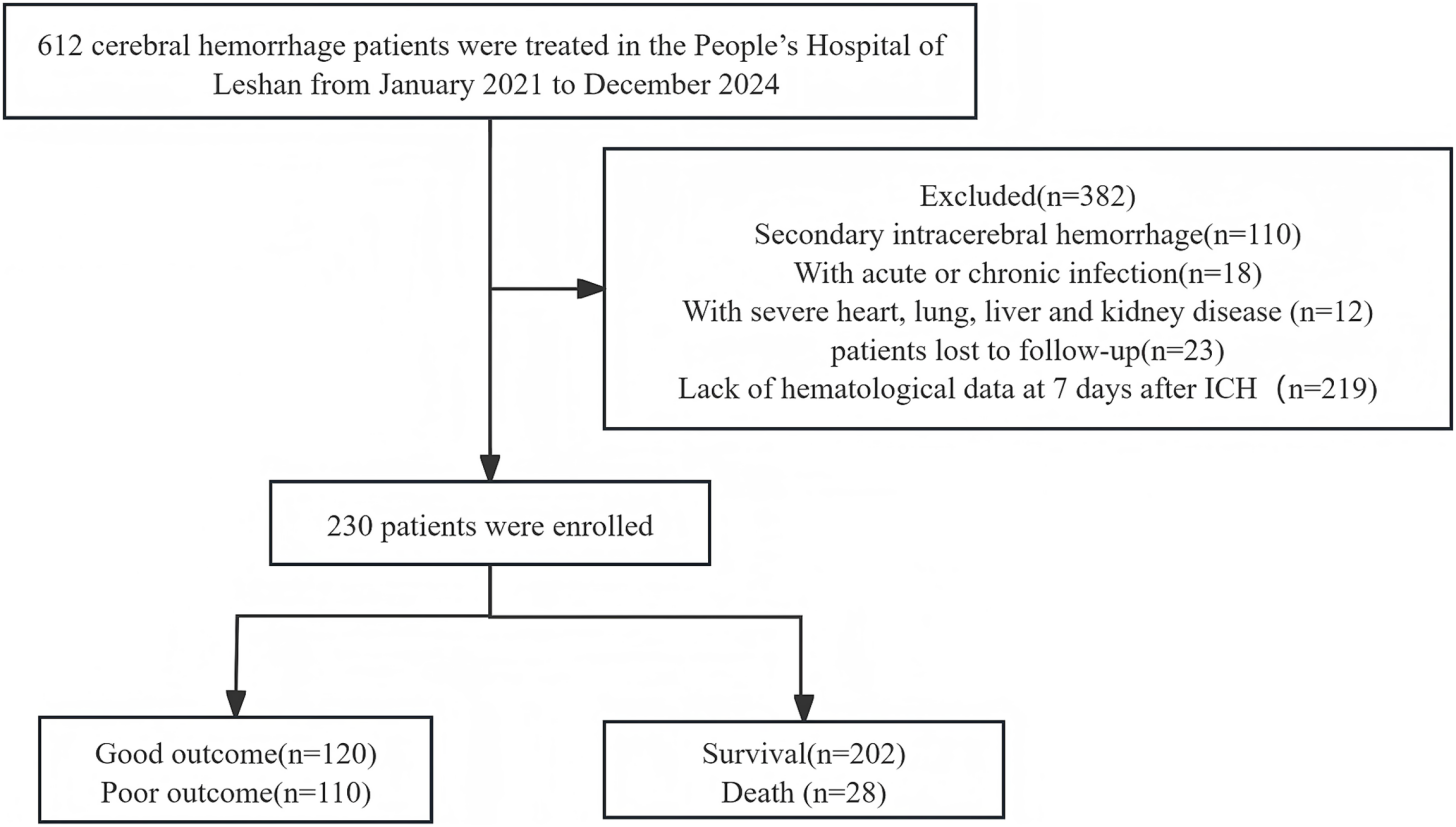
Flow diagram showing the patient selection process.

As this study aimed to evaluate the dynamic changes in inflammatory markers, only patients with both T1 and T2 hematological data were included in the final analysis. To assess potential selection bias, baseline characteristics were further compared between included patients and those excluded due to missing T2 data. The patient selection process is shown in Figure 1.

### Data collection

Clinical data were collected using a structured form by neurologists who had received standardized training. Baseline data included: (1) demographic characteristics, such as age and sex; (2) vascular risk factors and medical history, including hypertension, diabetes mellitus, atrial fibrillation, coronary artery disease, smoking history, and alcohol consumption; (3) prior medication use, including antiplatelet agents, anticoagulants, and statins; (4) vital signs and laboratory parameters at admission, including blood pressure, liver and renal function, and electrolytes; and (5) neurological status, assessed using the Glasgow Coma Scale (GCS). Neuroimaging data were obtained from head CT or MRI, including hematoma volume and the presence of intraventricular hemorrhage (IVH). In-hospital variables included pulmonary infection and surgical treatment status. Surgical treatment was recorded as a binary variable, but specific surgical procedures, such as craniotomy or minimally invasive surgery, were not further classified.

### Outcome assessment and definitions

Premorbid mRS scores were assessed by experienced clinicians based on information provided by patients or their family members regarding prior functional status. Only patients with premorbid mRS ≤ 2 were included. All patients were followed up at 90 days after onset via telephone interview. The 90-day mRS scores were independently assessed by two clinicians, and any discrepancies were resolved by consensus. The primary outcome was 90-day functional outcome, assessed using the mRS. Outcome classification was based on the absolute 90-day mRS score rather than change from premorbid mRS, with favorable outcome defined as mRS 0–2 and unfavorable outcome defined as mRS 3–6. The secondary outcome was 90-day mortality, defined as an mRS score of 6, whereas survival was defined as an mRS score of 0–5.

### Inflammatory marker detection and calculation formula

Venous blood samples were collected within 24 h after admission (T1) and at 7 days after onset (T2). If multiple complete blood count tests were performed within 24 h after admission, the earliest result was used as the T1 value. For T2 measurements, the result closest to day 7 after onset was used. The following laboratory parameters were recorded: neutrophil count (×10^9^/L), lymphocyte count (×10^9^/L), monocyte count (×10^9^/L), platelet count (×10^9^/L), high-sensitivity C-reactive protein (hs-CRP, mg/L), and serum albumin (ALB, g/L).

All cell counts were analyzed using absolute values obtained from routine blood tests. Based on previously reported definitions, the following composite inflammatory indices were calculated:

NLR = neutrophil count / lymphocyte count;

IPI = high-sensitivity C-reactive protein × neutrophil count / (lymphocyte count × serum albumin).

SII = platelet count × neutrophil count / lymphocyte count;

SIRI = monocyte count × neutrophil count / lymphocyte count.

To evaluate dynamic inflammatory burden, values of each inflammatory index at T1 and T2 were calculated. Fold change was calculated as FC = T2/T1, and relative percentage change was defined as c1–2 = (T2 − T1) / T1 × 100%, equivalent to (FC − 1) × 100%.

### Statistical analysis

Continuous variables were first tested for normality using the Shapiro–Wilk test. Variables with an approximately normal distribution were expressed as mean ± standard deviation (SD) and compared using the independent-samples t-test, whereas non-normally distributed variables were presented as median (interquartile range, IQR) and compared using the Wilcoxon rank-sum test (Mann–Whitney *U* test). Categorical variables were expressed as counts (percentages) and compared using the Pearson chi-square test or Fisher’s exact test when expected frequencies were <5. Dynamic changes in composite inflammatory indices, including the systemic immune-inflammation index (SII), systemic inflammation response index (SIRI), neutrophil-to-lymphocyte ratio (NLR), and inflammation prognostic index (IPI), between T1 and T2 were analyzed using the paired Wilcoxon signed-rank test for both overall and stratified analyses, and were visualized using line plots. Fold change was calculated as FC = T2/T1, and relative percentage change was defined as c1–2 = (T2 − T1) / T1 × 100%, equivalent to (FC − 1) × 100%. In regression analyses, c1–2 was divided by 10 before model entry; therefore, the odds ratio for c1–2 represents the effect per 10-percentage-point increase in relative change. To reduce skewness and facilitate interpretation of effect sizes, SII, SIRI, NLR, and IPI were log2-transformed and expressed as logT1 and logT2. Univariate logistic regression models were used to evaluate the associations of inflammatory indices with 90-day unfavorable functional outcomes and mortality. Multivariate logistic regression models were further constructed, adjusting for age, sex, admission Glasgow Coma Scale (GCS) score, hematoma volume, intraventricular hemorrhage (IVH), pulmonary infection, and surgical treatment. Discriminative performance was evaluated using receiver operating characteristic (ROC) curves and the area under the curve (AUC). Adjusted ROC curves were generated based on predicted probabilities from logistic regression models adjusted for age and sex. All statistical tests were two-sided, with a significance level of *α* = 0.05. Statistical analyses were performed using *R* software, version 4.3.2 (*R* Foundation for Statistical Computing, Vienna, Austria).

## Results

### Baseline characteristics

A total of 230 patients were included in this study, with a mean age of 61.92 ± 11.91 years. According to 90-day outcomes, patients were categorized into a favorable outcome group (*n* = 120) and an unfavorable outcome group (*n* = 110), and according to survival status, patients were categorized into a non-survivor group (*n* = 28) and a survivor group (*n* = 202). Compared with the favorable outcome group, patients with unfavorable outcomes had lower admission GCS scores, larger hematoma volumes, higher rates of intraventricular hemorrhage and pulmonary infection, and higher blood glucose levels at T1 (all *p* < 0.01). Levels of SII, SIRI, IPI, and NLR at both T1 and T2 were higher in the unfavorable outcome group, with more pronounced differences at T2 (all *p* < 0.001). No significant differences were observed between the groups in terms of sex, age, length of hospital stay, or proportion of surgical treatment (Table 1).

**Table 1:** Baseline characteristics according to 90-day functional outcomes.

Variable	Level	Total (*n* = 230)	Good (*n* = 120)	Poor (*n* = 110)	Statistic	*p*
Sex	Female	75 (32.61%)	41 (34.17%)	34 (30.91%)	χ^2^ = 0.15	0.700
	Male	155 (67.39%)	79 (65.83%)	76 (69.09%)		
Age		59.00 (54.00, 71.00)	60.99 ± 12.11	62.93 ± 11.67	t = −1.23	0.218
Admission GCS score		13.00 (9.00, 15.00)	14.00 (13.00, 15.00)	10.00 (5.00, 13.00)	Z = 8.81	<0.001
Length of hospital stay (days)		11.00 (7.00, 19.00)	11.00 (8.00, 16.00)	11.00 (6.00, 26.75)	Z = 0.40	0.686
Hematoma volume		14.00 (6.00, 27.00)	10.00 (4.38, 18.00)	24.00 (10.00, 40.00)	Z = 6.02	<0.001
Intraventricular hemorrhage (IVH)	No	150 (65.22%)	95 (79.17%)	55 (50.00%)	χ^2^ = 20.26	<0.001
	Yes	80 (34.78%)	25 (20.83%)	55 (50.00%)		
Pulmonary infection	No	172 (74.78%)	100 (83.33%)	72 (65.45%)	χ^2^ = 8.80	0.003
	Yes	58 (25.22%)	20 (16.67%)	38 (34.55%)		
Surgical treatment	No	107 (46.52%)	55 (45.83%)	52 (47.27%)	χ^2^ = 0.01	0.931
	Yes	123 (53.48%)	65 (54.17%)	58 (52.73%)		
Glucose (GLU)		6.54 (5.26, 8.58)	5.99 (4.91, 7.17)	7.39 (5.86, 9.90)	Z = 4.74	<0.001
Albumin (ALB) at T1		38.55 (35.82, 41.58)	39.07 ± 4.22	38.10 (34.17, 41.10)	Z = 1.81	0.070
Albumin (ALB) at T2		37.55 (32.92, 40.70)	39.66 ± 3.84	33.00 ± 5.71	t = 10.27	<0.001
SII at T1		1180.87 (618.76, 1979.36)	951.31 (513.14, 1663.91)	1526.70 (918.15, 2716.29)	Z = 4.17	<0.001
SII at T2		779.43 (375.74, 1736.98)	391.34 (260.07, 659.46)	1726.91 (962.29, 2818.02)	Z = 10.76	<0.001
SIRI at T1		2.53 (1.41, 5.01)	1.85 (1.15, 3.86)	3.42 (1.65, 8.41)	Z = 4.35	<0.001
SIRI at T2		1.95 (0.67, 5.43)	0.67 (0.38, 1.27)	5.80 (3.65, 9.93)	Z = 12.08	<0.001
IPI at T1		39.68 (12.08, 148.21)	24.28 (6.93, 90.32)	78.94 (24.54, 248.06)	Z = 4.27	<0.001
IPI at T2		22.05 (2.13, 272.97)	2.26 (0.73, 14.29)	280.64 (73.10, 784.96)	Z = 11.41	<0.001
NLR at T1		7.21 (3.85, 12.58)	5.65 (3.04, 9.65)	9.92 (5.87, 16.17)	Z = 4.67	<0.001
NLR at T2		3.67 (1.87, 8.03)	2.02 (1.31, 3.02)	8.15 (4.97, 12.12)	Z = 11.03	<0.001

In the mortality analysis, non-survivors had lower admission GCS scores, shorter hospital stays, larger hematoma volumes, and higher rates of pulmonary infection (all *p* < 0.05). In addition, blood glucose levels at T1 were higher in the non-survivors (*p* = 0.009). Inflammatory indices at T2 (SII, SIRI, IPI, and NLR) were significantly elevated (all *p* < 0.001), whereas most T1 indices did not show significant differences (Table 2).

**Table 2:** Baseline characteristics stratified by mortality.

Variable	Level	Total (*n* = 230)	Survivors (*n* = 202)	Non-survivors (*n* = 28)	Statistic	*p*
Sex	Female	75 (32.61%)	66 (32.67%)	9 (32.14%)	χ^2^ = 0.00	1.000
	Male	155 (67.39%)	136 (67.33%)	19 (67.86%)		
Age		59.00 (54.00, 71.00)	61.62 ± 11.88	62.50 (54.00, 75.25)	Z = 0.77	0.442
Admission GCS score		13.00 (9.00, 15.00)	14.00 (11.00, 15.00)	5.00 (4.00, 9.25)	Z = 5.96	<0.001
Length of hospital stay (days)		11.00 (7.00, 19.00)	12.00 (8.00, 19.00)	5.50 (2.75, 11.00)	Z = 3.97	<0.001
Hematoma volume		14.00 (6.00, 27.00)	13.25 (6.00, 25.00)	30.00 (11.50, 60.00)	Z = 3.46	<0.001
Intraventricular hemorrhage (IVH)	No	150 (65.22%)	136 (67.33%)	14 (50.00%)	χ^2^ = 2.54	0.111
	Yes	80 (34.78%)	66 (32.67%)	14 (50.00%)		
Pulmonary infection	No	172 (74.78%)	156 (77.23%)	16 (57.14%)	χ^2^ = 4.25	0.039
	Yes	58 (25.22%)	46 (22.77%)	12 (42.86%)		
Surgical treatment	No	107 (46.52%)	94 (46.53%)	13 (46.43%)	χ^2^ = 0.00	1.000
	Yes	123 (53.48%)	108 (53.47%)	15 (53.57%)		
Glucose (GLU)		6.54 (5.26, 8.58)	6.27 (5.14, 8.28)	7.27 (6.04, 11.08)	Z = 2.60	0.009
Albumin (ALB) at T1		38.55 (35.82, 41.58)	38.65 (35.82, 41.68)	38.01 ± 5.44	Z = 0.06	0.954
Albumin (ALB) at T2		37.55 (32.92, 40.70)	38.30 (34.10, 40.98)	30.33 ± 5.95	Z = 5.29	<0.001
SII at T1		1180.87 (618.76, 1979.36)	1156.20 (607.04, 1943.00)	1453.59 (957.31, 2318.98)	Z = 1.18	0.237
SII at T2		779.43 (375.74, 1736.98)	647.94 (336.48, 1374.60)	3279.22 ± 1480.83	Z = 7.08	<0.001
SIRI at T1		2.53 (1.41, 5.01)	2.42 (1.41, 4.58)	4.78 (1.68, 8.81)	Z = 1.90	0.058
SIRI at T2		1.95 (0.67, 5.43)	1.54 (0.57, 4.33)	9.93 (7.67, 13.27)	Z = 6.99	<0.001
IPI at T1		39.68 (12.08, 148.21)	37.83 (11.78, 148.21)	75.06 (19.01, 155.81)	Z = 0.95	0.344
IPI at T2		22.05 (2.13, 272.97)	14.54 (1.58, 122.37)	884.12 (463.33, 1507.17)	Z = 7.13	<0.001
NLR at T1		7.21 (3.85, 12.58)	6.86 (3.77, 12.46)	9.98 (5.52, 15.01)	Z = 1.69	0.092
NLR at T2		3.67 (1.87, 8.03)	3.04 (1.67, 6.40)	13.20 ± 6.42	Z = 6.61	<0.001

*Continuous variables are presented as mean ± standard deviation or median (interquartile range), as appropriate. For variables with different distribution patterns across subgroups, descriptive formats may differ between columns. Between-group comparisons were performed using the test shown in the Statistic column, where t indicates the independent-samples t-test and Z indicates the Wilcoxon rank-sum test. Categorical variables are presented as n (%).

### Comparison between included and excluded patients

There were no statistically significant differences between the included group (n = 230) and the excluded group (*n* = 219) in baseline characteristics, including sex, age, hematoma volume, admission GCS score, intraventricular hemorrhage, pulmonary infection, alcohol consumption history, hypertension, diabetes mellitus, antiplatelet therapy, and surgical treatment (all *p* > 0.05). However, the excluded group had a shorter length of hospital stay (*p* < 0.001; Table 3).

**Table 3:** Baseline characteristics of included and excluded patients.

Variable	Level	Included (*n* = 230)	Excluded (*n* = 219)	Statistic	*p* value
Sex	Female	75 (32.61%)	76 (34.70%)	χ^2^ = 0.22	0.639
	Male	155 (67.39%)	143 (65.30%)		
Age		59.00 (54.00, 71.00)	59.00 (52.00, 71.00)	Z = 0.51	0.607
Length of hospital stay (days)		11.00 (7.00, 19.00)	8.00 (5.00, 12.00)	Z = 5.57	<0.001
Hematoma volume		14.00 (6.00, 27.00)	13.00 (6.22, 27.00)	Z = 0.10	0.923
Intraventricular hemorrhage (IVH)	No	150 (65.22%)	141 (64.38%)	χ^2^ = 0.03	0.853
	Yes	80 (34.78%)	78 (35.62%)		
Admission GCS score		13.00 (9.00, 15.00)	14.00 (5.00, 15.00)	Z = 0.23	0.820
Pulmonary infection	No	172 (74.78%)	158 (72.15%)	χ^2^ = 0.40	0.527
	Yes	58 (25.22%)	61 (27.85%)		
Alcohol consumption	No	152 (66.09%)	155 (70.78%)	χ^2^ = 1.14	0.285
	Yes	78 (33.91%)	64 (29.22%)		
Hypertension	No	24 (10.43%)	32 (14.61%)	χ^2^ = 1.79	0.181
	Yes	206 (89.57%)	187 (85.39%)		
Diabetes mellitus	No	208 (90.43%)	194 (88.58%)	χ^2^ = 0.41	0.522
	Yes	22 (9.57%)	25 (11.42%)		
Antiplatelet therapy	No	223 (96.96%)	210 (95.89%)	χ^2^ = 0.37	0.542
	Yes	7 (3.04%)	9 (4.11%)		
Surgical treatment	No	107 (46.52%)	115 (52.51%)	χ^2^ = 1.61	0.204
	Yes	123 (53.48%)	104 (47.49%)		

### Dynamic changes in inflammatory indices

**Figure 2:**
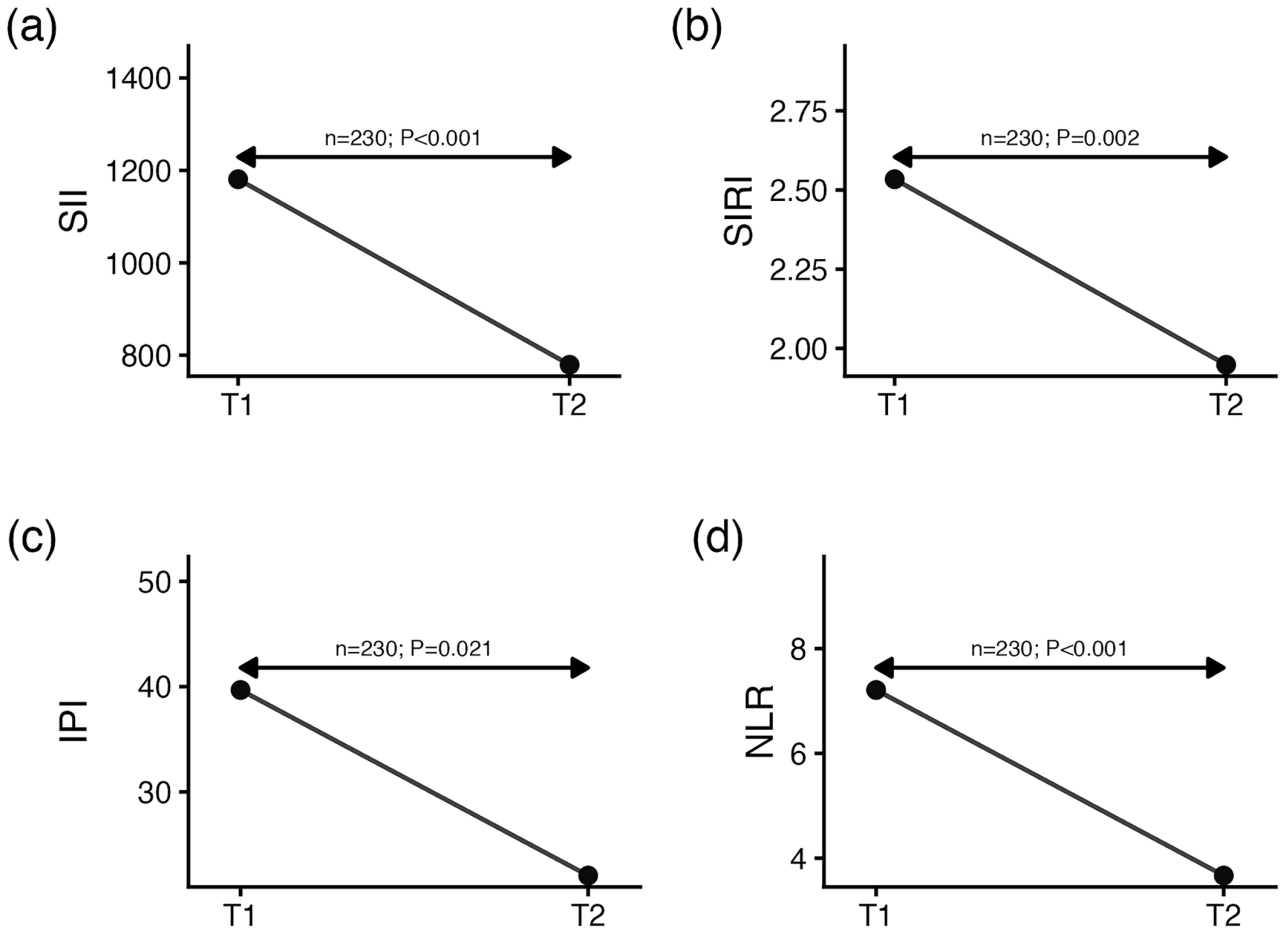
Dynamic changes in composite inflammatory indices from T1 to T2. **(a)** SII; **(b)** SIRI; **(c)** IPI; **(d)** NLR. *p* values from the Wilcoxon signed-rank test. Abbreviations: SII, systemic immune-inflammation index; SIRI, systemic inflammation response index; IPI, inflammation prognostic index; NLR, neutrophil-to-lymphocyte ratio; T1, within 24 h of admission; T2, day 7 after onset.

**Figure 3:**
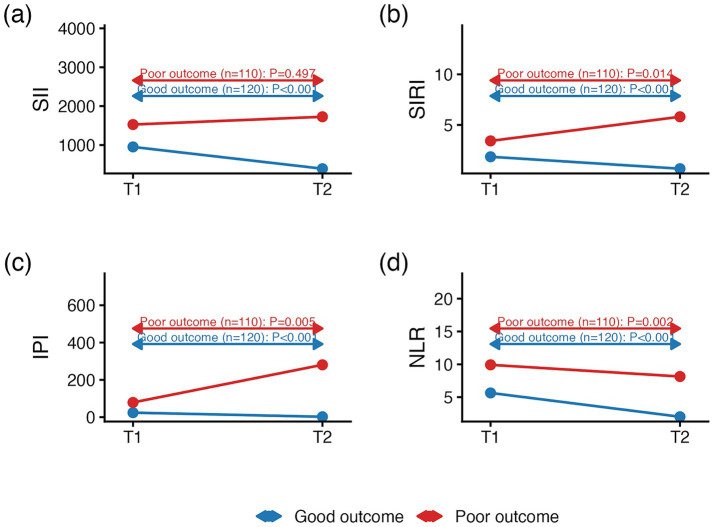
T1–T2 changes in inflammatory indices by 90-day functional outcome (good vs. poor). **(a)** SII; **(b)** SIRI; **(c)** IPI; **(d)** NLR. *p* values from the Wilcoxon signed-rank test. Abbreviations as in Figure 2.

**Figure 4:**
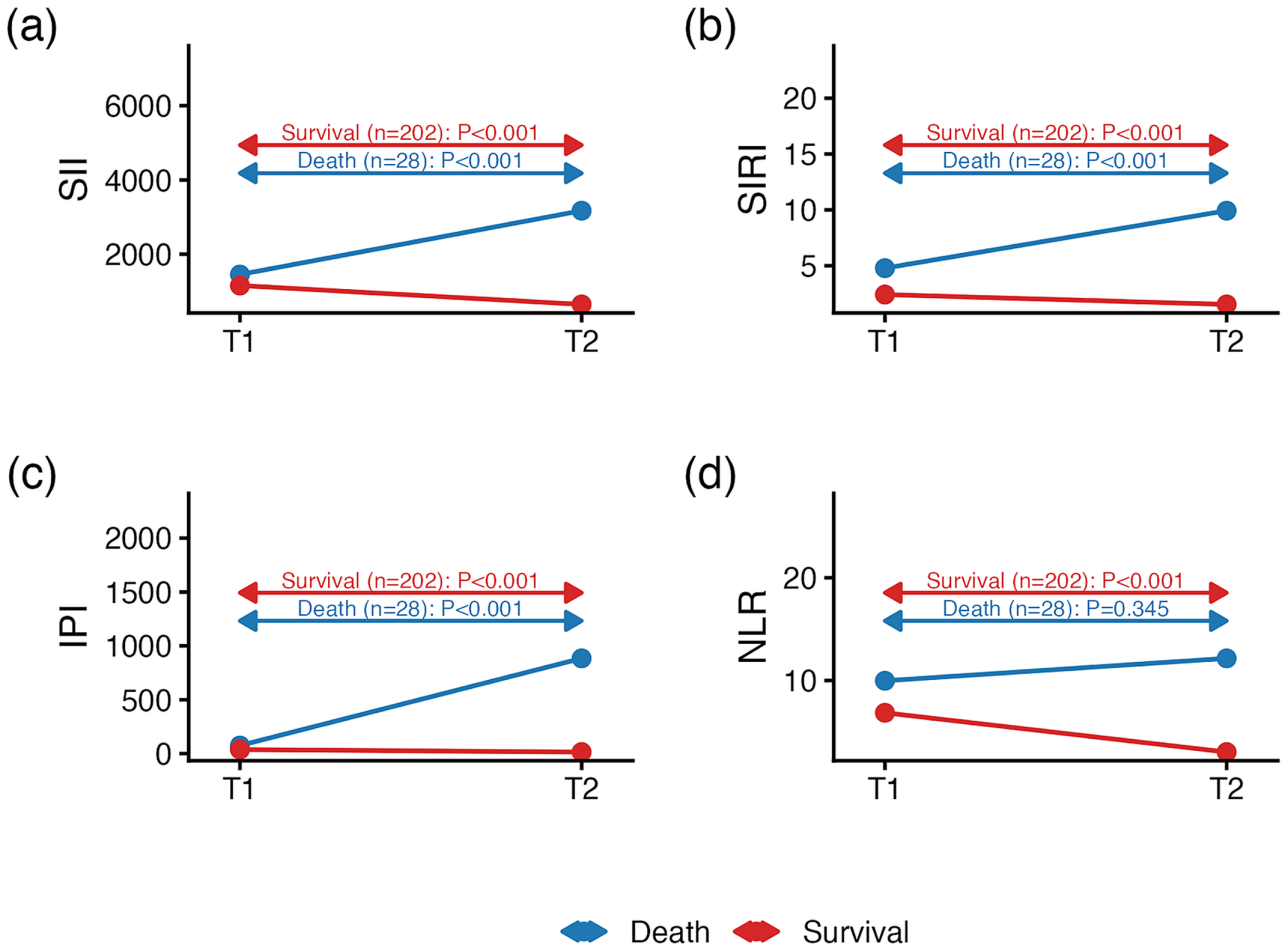
T1–T2 changes in inflammatory indices by 90-day survival (survivors vs. non-survivors). **(a)** SII; **(b)** SIRI; **(c)** IPI; **(d)** NLR. *p* values from the Wilcoxon signed-rank test. Abbreviations as in Figure 2.

Line plots of overall dynamic changes in inflammatory indices (SII, SIRI, NLR, and IPI) from T1 to T2 showed an overall decreasing trend, with all changes reaching statistical significance (*p* < 0.05; Figure 2). When stratified by 90-day functional outcome or survival status, most indices showed greater changes in patients with unfavorable outcomes and in non-survivors, who generally exhibited increasing trends, whereas patients with favorable outcomes and survivors showed significant decreasing trends (Figures 3, 4), except for SII in the unfavorable outcome group and NLR in non-survivors.

Supplementary Figures S1–S4 illustrate the distribution of percentage changes (c1–2) in IPI, NLR, SII, and SIRI from T1 to T2, stratified by 90-day outcomes and survival status. Boxplot analysis showed that the magnitude of c1–2 changes was greater in the unfavorable outcome and non-survivors compared with patients with favorable outcomes and survivors, respectively.

### Univariate logistic regression analysis

For mortality, all four inflammatory indices at T2 were significantly positively associated with the risk of death: IPI logT2 (OR = 2.10, *p* < 0.001), NLR logT2 (OR = 4.90, *p* < 0.001), SII logT2 (OR = 5.74, *p* < 0.001), and SIRI logT2 (OR = 4.08, *p* < 0.001). Regarding dynamic changes, expressed as c1–2 per 10-percentage-point increase, NLR c1–2 (OR = 1.04, *p* = 0.003), SII c1–2 (OR = 1.03, *p* < 0.001), and SIRI c1–2 (OR = 1.02, *p* = 0.002) were significantly associated with mortality, whereas IPI c1–2 did not reach statistical significance (*p* = 0.072). None of the four indices at T1 were significantly associated with mortality (all *p* > 0.05). Among baseline variables, length of hospital stay was negatively associated with mortality (OR = 0.95, *p* = 0.044), whereas hematoma volume was positively associated (OR = 1.04, *p* < 0.001). Alcohol consumption (OR = 3.01, *p* = 0.007), antiplatelet therapy (OR = 5.94, *p* = 0.025), and pulmonary infection (OR = 3.59, *p* = 0.008) were also associated with increased mortality risk. For 90-day unfavorable functional outcomes, all four inflammatory indices at T2 were significantly associated with increased odds of unfavorable functional outcomes: IPI logT2 (OR = 2.03, *p* < 0.001), NLR logT2 (OR = 7.31, *p* < 0.001), SII logT2 (OR = 5.65, *p* < 0.001), and SIRI logT2 (OR = 7.52, *p* < 0.001). In addition, all four dynamic change indicators, expressed as c1–2 per 10-percentage-point increase, were significantly associated with unfavorable outcomes: IPI c1–2 (OR = 1.18, *p* < 0.001), NLR c1–2 (OR = 1.24, *p* < 0.001), SII c1–2 (OR = 1.21, *p* < 0.001), and SIRI c1–2 (OR = 1.31, *p* < 0.001). Unlike mortality, all four indices at T1 were also significantly associated with unfavorable outcomes (all *p* < 0.001). Among baseline variables, length of hospital stay (OR = 1.02, *p* = 0.019), hematoma volume (OR = 1.06, *p* < 0.001), intraventricular hemorrhage (OR = 3.80, *p* < 0.001), other comorbid conditions (OR = 3.54, *p* < 0.001), pulmonary infection (OR = 5.44, *p* < 0.001), and venous thrombosis (OR = 2.44, *p* = 0.030) were associated with increased risk of unfavorable outcomes (Table 4).

**Table 4:** Univariate logistic regression analysis.

Variable	Death			Poor outcome		
	OR	95% CI	*p*	OR	95% CI	*p*
Age	1.02	0.98, 1.05	0.316	1.01	0.99, 1.04	0.219
Heart rate	0.99	0.96, 1.02	0.507	1.02	1.00, 1.04	0.059
Systolic blood pressure (SBP)	0.99	0.97, 1.01	0.352	1.01	0.99, 1.02	0.349
Diastolic blood pressure (DBP)	0.99	0.96, 1.01	0.280	1	0.99, 1.02	0.713
Length of hospital stay (days)	0.95	0.90, 0.99	0.044	1.02	1.01, 1.05	0.019
Hematoma volume	1.04	1.02, 1.06	<0.001	1.06	1.04, 1.08	<0.001
Sex						
Female	Ref.	Ref.		Ref.	Ref.	
Male	1.02	0.45, 2.49	0.955	1.16	0.67, 2.02	0.599
Intraventricular hemorrhage (IVH)						
No	Ref.	Ref.		Ref.	Ref.	
Yes	2.06	0.92, 4.61	0.075	3.8	2.15, 6.86	<0.001
Hypertension						
No	Ref.	Ref.		Ref.	Ref.	
Yes	0.97	0.30, 4.30	0.959	1.32	0.56, 3.19	0.524
Diabetes mellitus						
No	Ref.	Ref.		Ref.	Ref.	
Yes	0.7	0.11, 2.59	0.643	1.65	0.68, 4.17	0.270
Heart disease						
No	Ref.	Ref.		Ref.	Ref.	
Yes	2.91	0.61, 10.8	0.132	1.33	0.39, 4.73	0.648
Kidney disease						
No	Ref.	Ref.		Ref.	Ref.	
Yes	0.79	0.04, 4.48	0.830	2.65	0.72, 12.5	0.166
Smoking history						
No	Ref.	Ref.		Ref.	Ref.	
Yes	1.91	0.86, 4.34	0.112	1.21	0.72, 2.04	0.480
Alcohol use history						
No	Ref.	Ref.		Ref.	Ref.	
Yes	3.01	1.35, 6.87	0.007	1.44	0.83, 2.50	0.191
Family history						
No	Ref.	Ref.		Ref.	Ref.	
Yes	3.7	0.17, 39.9	0.292	0.54	0.02, 5.73	0.618
Antiplatelet therapy						
No	Ref.	Ref.		Ref.	Ref.	
Yes	5.94	1.12, 28.5	0.025	1.47	0.32, 7.61	0.618
Other comorbidities						
No	Ref.	Ref.		Ref.	Ref.	
Yes	2.22	0.97, 4.99	0.054	3.54	1.93, 6.71	<0.001
Epilepsy						
No	Ref.	Ref.		Ref.	Ref.	
Yes	2.91	0.61, 10.8	0.132	1.97	0.58, 7.70	0.290
Pulmonary infection						
No	Ref.	Ref.		Ref.	Ref.	
Yes	3.59	1.48, 10.1	0.008	5.44	3.11, 9.74	<0.001
Venous thrombosis						
No	Ref.	Ref.		Ref.	Ref.	
Yes	1.54	0.48, 4.15	0.423	2.44	1.11, 5.69	0.030
IPI logT1	1.05	0.92, 1.19	0.49	1.21	1.10, 1.33	<0.001
IPI logT2	2.1	1.63, 2.90	<0.001	2.03	1.73, 2.45	<0.001
IPI c1–2	1	1.00, 1.00	0.072	1.18	1.11, 1.27	<0.001
NLR logT1	1.33	0.94, 1.91	0.111	1.74	1.37, 2.25	<0.001
NLR logT2	4.9	2.92, 9.29	<0.001	7.31	4.69, 12.4	<0.001
NLR c1–2	1.04	1.02, 1.07	0.003	1.24	1.15, 1.35	<0.001
SII logT1	1.23	0.89, 1.73	0.23	1.59	1.27, 2.02	<0.001
SII logT2	5.74	3.32, 11.4	<0.001	5.65	3.82, 8.95	<0.001
SII c1–2	1.03	1.01, 1.05	<0.001	1.21	1.13, 1.30	<0.001
SIRI logT1	1.29	0.98, 1.72	0.068	1.54	1.26, 1.90	<0.001
SIRI logT2	4.08	2.55, 7.41	<0.001	7.52	4.70, 13.6	<0.001
SIRI c1–2	1.02	1.01, 1.03	0.002	1.31	1.21, 1.44	<0.001

### Multivariate analysis

In the models for 90-day unfavorable functional outcomes, T2 levels and c1–2 indicators of all four inflammatory indices remained significantly associated with increased odds of unfavorable outcomes after adjustment. In the mortality models, T2 levels of all four inflammatory indices remained significantly associated with 90-day mortality after adjustment. Among the dynamic change indicators, IPI c1–2, SII c1–2, and SIRI c1–2 remained significant, whereas NLR c1–2 showed a borderline association. The inverse association between IPI logT1 and mortality should be interpreted cautiously, possibly reflecting model instability or residual confounding (Table 5).

**Table 5:** Multivariable logistic regression analysis.

Predictor	Poor outcome			Death		
	OR(95%CI)	*p*	Events (*n*)	OR(95%CI)	*p*	Events (*n*)
IPI logT1	1.00 (0.88–1.13)	0.982	110	0.80 (0.66–0.97)	0.023	28
IPI logT2	1.95 (1.57–2.42)	<0.001	110	1.97 (1.40–2.76)	<0.001	28
IPI c1–2	1.12 (1.05–1.20)	0.001	110	1.00 (1.00–1.01)	0.033	28
NLR logT1	1.16 (0.85–1.58)	0.343	110	0.79 (0.51–1.21)	0.278	28
NLR logT2	5.16 (2.98–8.93)	<0.001	110	3.62 (1.75–7.49)	<0.001	28
NLR c1–2	1.17 (1.09–1.26)	<0.001	110	1.03 (1.00–1.05)	0.062	28
SII logT1	1.20 (0.90–1.60)	0.206	110	0.82 (0.55–1.21)	0.309	28
SII logT2	4.06 (2.54–6.50)	<0.001	110	4.78 (2.30–9.92)	<0.001	28
SII c1–2	1.14 (1.07–1.23)	<0.001	110	1.02 (1.00–1.04)	0.033	28
SIRI logT1	1.08 (0.83–1.40)	0.590	110	0.81 (0.57–1.16)	0.255	28
SIRI logT2	9.85 (4.41–21.98)	<0.001	110	3.55 (1.84–6.84)	<0.001	28
SIRI c1–2	1.26 (1.14–1.40)	<0.001	110	1.01 (1.00–1.02)	0.042	28

*logT1 and logT2 represent log2-transformed values at T1 and T2. c1–2 = (T2 − T1)/T1 × 100%, and c1–2 was divided by 10 before entering the model. The OR for log2-transformed markers represents the effect per doubling (×2) increase; the OR for c1–2 represents the effect per 10-percentage-point increase in relative change. OR = odds ratio.

### Sensitivity analysis

Sensitivity analyses further adjusting for glucose (GLU) and albumin (ALB) showed consistent results, with no substantial changes in the direction of associations (Table 6).

**Table 6:** Sensitivity analysis for 90-day outcomes.

Predictor	Poor outcome OR(95%CI)	*p* value	Events (*n*)	Death OR(95%CI)	*p* value	*n*
IPI logT1	0.95 (0.82–1.10)	0.495	110	0.80 (0.64–0.99)	0.042	28
IPI logT2	1.84 (1.47–2.30)	<0.001	110	1.89 (1.33–2.68)	<0.001	28
IPI c1–2	1.12 (1.04–1.20)	0.003	110	1.00 (1.00–1.00)	0.031	28
NLR logT1	1.04 (0.74–1.47)	0.811	110	0.73 (0.45–1.18)	0.196	28
NLR logT2	4.54 (2.55–8.09)	<0.001	110	3.54 (1.59–7.87)	0.002	28
NLR c1–2	1.17 (1.08–1.27)	<0.001	110	1.03 (1.00–1.05)	0.050	28
SII logT1	1.12 (0.81–1.54)	0.487	110	0.76 (0.49–1.17)	0.209	28
SII logT2	3.65 (2.22–5.99)	<0.001	110	6.23 (2.60–14.92)	<0.001	28
SII c1–2	1.14 (1.06–1.22)	<0.001	110	1.02 (1.00–1.04)	0.018	28
SIRI logT1	1.04 (0.78–1.38)	0.811	110	0.77 (0.52–1.14)	0.193	28
SIRI logT2	10.19 (4.31–24.08)	<0.001	110	4.45 (1.95–10.13)	<0.001	28
SIRI c1–2	1.27 (1.13–1.43)	<0.001	110	1.01 (1.00–1.02)	0.027	28

*Abbreviations and variable transformations are as described in Table 5.

### Predictive performance of inflammatory indices

ROC analysis demonstrated good discriminative performance of all inflammatory indices for predicting 90-day unfavorable outcomes and mortality, with T2 indices generally outperforming T1 indices. Among them, SIRI logT2 showed the highest AUC for unfavorable outcomes, while multiple T2 indices also exhibited strong predictive performance for mortality (Table 7).

**Table 7:** Adjusted ROC analysis for 90-day unfavorable functional outcomes and mortality.

Predictor	Poor AUC (95% CI)	Cut-off	Sensitivity	Specificity	*n*	Death AUC (95% CI)	Cut-off	Sensitivity	Specificity	*n*
IPI logT1	0.869 (0.816–0.914)	0.465	0.800	0.817	110	0.865 (0.778–0.956)	0.219	0.786	0.891	28
IPI logT2	0.945 (0.915–0.970)	0.460	0.882	0.867	110	0.935 (0.900–0.967)	0.105	1.000	0.807	28
IPI c1–2	0.938 (0.907–0.964)	0.556	0.791	0.950	110	0.891 (0.825–0.950)	0.205	0.786	0.891	28
NLR logT1	0.870 (0.820–0.916)	0.486	0.791	0.833	110	0.868 (0.787–0.949)	0.182	0.786	0.851	28
NLR logT2	0.936 (0.904–0.961)	0.725	0.755	0.967	110	0.915 (0.870–0.952)	0.051	1.000	0.708	28
NLR c1–2	0.905 (0.867–0.938)	0.436	0.818	0.833	110	0.888 (0.824–0.949)	0.200	0.786	0.896	28
SII logT1	0.872 (0.823–0.916)	0.384	0.873	0.742	110	0.872 (0.793–0.949)	0.189	0.786	0.866	28
SII logT2	0.934 (0.904–0.961)	0.694	0.764	0.933	110	0.936 (0.890–0.967)	0.200	0.857	0.906	28
SII c1–2	0.906 (0.865–0.937)	0.510	0.791	0.883	110	0.895 (0.837–0.951)	0.205	0.786	0.901	28
SIRI logT1	0.872 (0.821–0.915)	0.463	0.809	0.808	110	0.873 (0.802–0.946)	0.197	0.786	0.866	28
SIRI logT2	0.967 (0.949–0.983)	0.269	0.973	0.817	110	0.927 (0.890–0.963)	0.049	1.000	0.723	28
SIRI c1–2	0.936 (0.906–0.962)	0.397	0.845	0.883	110	0.896 (0.839–0.953)	0.195	0.786	0.886	28

Pairwise comparisons of AUC values showed that T2 indices outperformed T1 indices in most comparisons, and some dynamic change indicators (c1–2) also showed better performance than baseline measures (Table 8).

**Table 8:** Comparison of AUC values of different forms of the same indicator for 90-day outcomes.

Model1	Model2	Poor outcome AUC1	AUC2	*p* value	Death AUC1	AUC2	*p* value
IPI logT1	IPI logT2	0.869	0.945	<0.001	0.865	0.935	0.067
IPI logT1	IPI c1–2	0.869	0.938	<0.001	0.865	0.891	0.124
IPI logT2	IPI c1–2	0.945	0.938	0.568	0.935	0.891	0.088
NLR logT1	NLR logT2	0.870	0.936	<0.001	0.868	0.915	0.123
NLR logT1	NLR c1–2	0.870	0.905	0.027	0.868	0.888	0.059
NLR logT2	NLR c1–2	0.936	0.905	0.019	0.915	0.888	0.212
SII logT1	SII logT2	0.872	0.934	<0.001	0.872	0.936	0.063
SII logT1	SII c1–2	0.872	0.906	0.026	0.872	0.895	0.063
SII logT2	SII c1–2	0.934	0.906	0.031	0.936	0.895	0.113
SIRI logT1	SIRI logT2	0.872	0.967	<0.001	0.873	0.927	0.060
SIRI logT1	SIRI c1–2	0.872	0.936	<0.001	0.873	0.896	0.077
SIRI logT2	SIRI c1–2	0.967	0.936	0.013	0.927	0.896	0.142

ROC curves are presented in Supplementary Figures S5–S12. Overall, the four composite inflammatory indices at T2 and the dynamic change indicators from T1 to T2 showed good discriminative ability for predicting 90-day unfavorable outcomes and mortality, with AUC values generally higher than those of baseline T1 measurements. After adjustment for age and sex, AUC values were further improved, suggesting that day-7 inflammatory status and its dynamic changes may provide more prognostic information than single baseline measurements.

## Discussion

The findings of the present study further support the important role of inflammatory responses in determining the prognosis of ICH. Unlike previous studies that mainly focused on inflammatory markers at a single time point, this study evaluated the prognostic value of multiple composite inflammatory indices from a temporal perspective.

From a pathophysiological perspective, inflammatory cascades are considered to be involved in the development of secondary brain injury following ICH (21). After intracerebral hemorrhage, neutrophils are among the earliest peripheral immune cells to be mobilized and infiltrate the perihematomal brain tissue (22). Experimental studies have suggested that mediators released by neutrophils, including proteolytic enzymes, reactive oxygen species, and matrix metalloproteinases, may be associated with blood–brain barrier disruption and perihematomal edema formation, thereby contributing to enhanced local inflammatory responses and secondary brain injury (21, 23). Correspondingly, inhibition of neutrophil activation or reduction of neutrophil infiltration has been associated with milder early brain edema and less severe neurological impairment, suggesting that neutrophils may participate in the process of secondary injury after ICH (24).

In addition to local inflammatory activation, ICH may also induce a systemic immune imbalance characterized by peripheral lymphopenia and immunosuppression (25, 26). Studies have shown that, following ICH, activation of the sympathetic nervous system and the hypothalamic–pituitary–adrenal axis is accompanied by increased peripheral lymphocyte apoptosis and a shift in the Th1/Th2 immune balance toward Th2 dominance (27). To some extent, this immunosuppressive state may be associated with limitation of excessive inflammatory responses; however, it may also impair host defense against infection, thereby increasing the risk of secondary infection and indirectly affecting patient prognosis through a “second-hit” mechanism (28).

Albumin is generally considered to play a protective role in the pathological process following ICH (29). Previous studies have shown that albumin not only maintains colloid osmotic pressure, but also exerts antioxidant and anti-inflammatory effects and helps stabilize the vascular endothelial barrier. By attenuating oxidative stress and inflammation-related blood–brain barrier damage and improving cerebral microcirculatory perfusion, albumin may be associated with less severe brain edema and reduced secondary brain injury (30–32). Therefore, hypoalbuminemia in patients with ICH may reflect not only poor nutritional status, but also a complex pathological state characterized by enhanced inflammatory responses and impaired vascular barrier function.

As a classical acute-phase reactant, high-sensitivity C-reactive protein (hs-CRP) reflects the level of systemic inflammatory activation driven by inflammatory cytokines. Elevated hs-CRP is closely associated with neutrophil activation, endothelial dysfunction, and complement activation, and may participate in amplification of the inflammatory cascade after ICH, thereby contributing to perihematomal edema and unfavorable outcomes (33–35).

In addition to their role in hemostasis, platelets may also participate in the process of secondary injury after hemorrhagic stroke through inflammation-related pathways (36). Studies have shown that platelet activation after ICH may act synergistically with inflammatory pathways and may be associated with increased immune cell recruitment, aggravated brain edema, and blood–brain barrier damage (37, 38). Platelet-derived growth factors, such as PDGF, are also considered to be involved in vascular remodeling and inflammatory regulation, further supporting the multifaceted role of platelets in the pathological process of ICH (39).

Taken together, neutrophil-related local inflammatory injury, immunosuppressive status reflected by lymphocyte reduction, attenuation of the protective properties of albumin, and the intensity of systemic inflammation reflected by hs-CRP jointly constitute an important pathobiological background for secondary brain injury after ICH.

Against this mechanistic background, the present study evaluated the relationship between composite inflammatory indices and ICH prognosis. The results showed that, after adjustment for clinically important factors including age, sex, GCS score, hematoma volume, intraventricular hemorrhage (IVH), infection, and surgical treatment, inflammatory marker levels at day 7 after onset (T2) and their dynamic changes (c1–2) remained significantly associated with 90-day unfavorable functional outcomes and mortality, whereas baseline (T1) levels did not show independent associations in most models. These findings indicate a clear time-dependent pattern in the prognostic value of inflammatory responses.

At baseline (T1), although composite inflammatory indices were generally higher in patients with unfavorable outcomes and in those who died, their discriminative ability was relatively limited. In contrast, inflammatory indices at T2 showed more stable predictive performance across analyses, suggesting that persistent or elevated inflammatory status may be more closely associated with unfavorable outcomes.

The magnitude of change in inflammatory indices from T1 to T2 also showed good discriminative ability, indicating that the dynamic evolution of inflammatory responses itself contains important prognostic information. These changes may be influenced by disease progression, treatment response, and in-hospital complications, and thus may better reflect the overall disease course than baseline status alone.

The present study also found that dynamic inflammatory indices were more prominently associated with mortality risk. Compared with unfavorable functional outcomes, mortality risk showed a weaker association with baseline inflammatory levels and was more strongly reflected by changes in inflammatory status during the first week of hospitalization. This suggests that later inflammatory imbalance and related complications, such as infection, may be more closely associated with mortality risk.

Although important clinical variables were adjusted for in the multivariable models, the observed associations between inflammatory indices and prognosis may still partly reflect the combined effects of initial ICH severity and in-hospital complications. Therefore, their independent predictive value should be interpreted with caution.

Patients included in the analysis were required to have day 7 hematological test results. Some patients did not complete day 7 testing because of early death, early discharge, or transfer, and the excluded group had a shorter length of hospital stay. Although baseline characteristics were comparable between included and excluded patients, selection bias and survivorship bias cannot be fully excluded. Therefore, the findings are mainly applicable to patients who completed T2 measurements.

Some inflammatory indices showed relatively high AUC values in ROC analysis. However, given that this was a single-center retrospective study with a limited sample size, these findings still require validation in larger multicenter studies.

This study included multiple composite inflammatory indices and conducted a systematic comparison, which may reduce bias associated with single-marker selection. By incorporating dynamic changes between T1 and T2, inflammatory responses were evaluated from a temporal perspective, complementing traditional static analyses and enhancing the robustness of the findings.

This study has several limitations. As a single-center retrospective analysis, selection bias and information bias may exist. Patients were required to have both T1 and T2 data, and some were excluded due to early death or discharge, which may introduce non-random missingness. Inflammatory markers were measured at only two time points and may not fully capture long-term dynamics. Residual confounding cannot be excluded, and no stratified analysis by hemorrhage location or surgical subtype was performed. Multiple comparisons and a relatively small number of death events also require cautious interpretation. As an observational study, causal relationships cannot be established.

In summary, composite inflammatory indices derived from routine blood parameters may have potential value in prognostic assessment of ICH, exhibiting a clear time-dependent pattern. Dynamic inflammatory indices show greater stability and discriminative ability compared with single time-point measurements and may serve as useful complements to conventional clinical indicators.

## Conclusion

The present findings suggest that composite inflammatory indices derived from routine blood test parameters may have potential value in prognostic assessment in patients with spontaneous intracerebral hemorrhage, and that their discriminative performance exhibits a clear time-dependent pattern. Compared with a single baseline measurement at admission, inflammatory levels at day 7 after onset (T2) and their dynamic changes from T1 to T2 showed more stable associations with unfavorable functional outcomes and 90-day mortality, and demonstrated better discriminative ability. Dynamic inflammatory features may serve as useful complements to conventional clinical indicators and provide a simple and feasible reference for risk stratification and follow-up management in patients during the acute phase.

## Data Availability

The raw data supporting the conclusions of this article will be made available by the authors, without undue reservation.
