# Supplementary material for: Dynamic inflammatory markers as predictors of 90-day outcomes in spontaneous intracerebral hemorrhage
Source: Front Neurol. 2026 Jun 18;17:1787030. doi: 10.3389/fneur.2026.1787030 (PMC13322834; doi:10.3389/fneur.2026.1787030)
Supplement: Supplementary file 1 [file Table_1.DOCX]

**
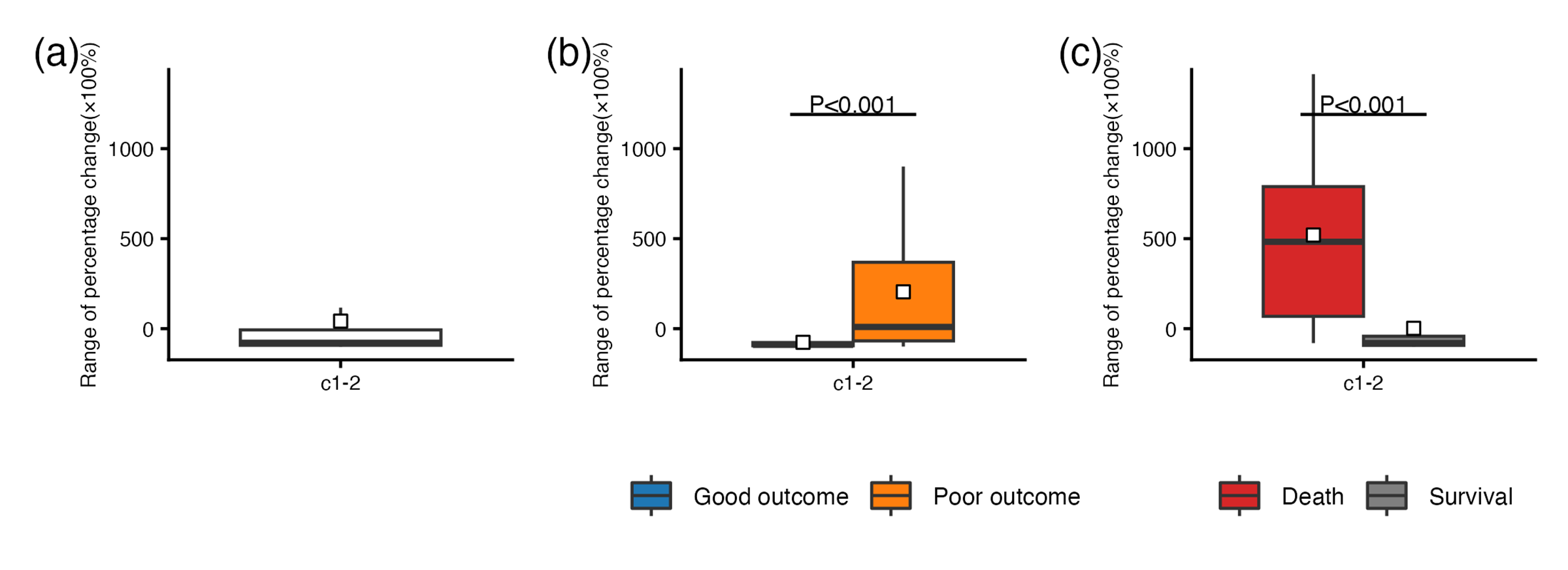
**Figure** S1.** Percentage changes in IPI from T1 to T2: **(a) Overall distribution; (b) stratified by 3-month functional outcome (good vs poor); (c) stratified by 3-month survival (survivors vs deaths)**. White squares = means, boxes = interquartile ranges(IQRs), central lines = medians. P values from Wilcoxon rank-sum test; c1–2 denotes T1→T2. Abbreviations: IPI, inflammation prognostic index; IQR, interquartile range; T1/T2 as in Figure 2.


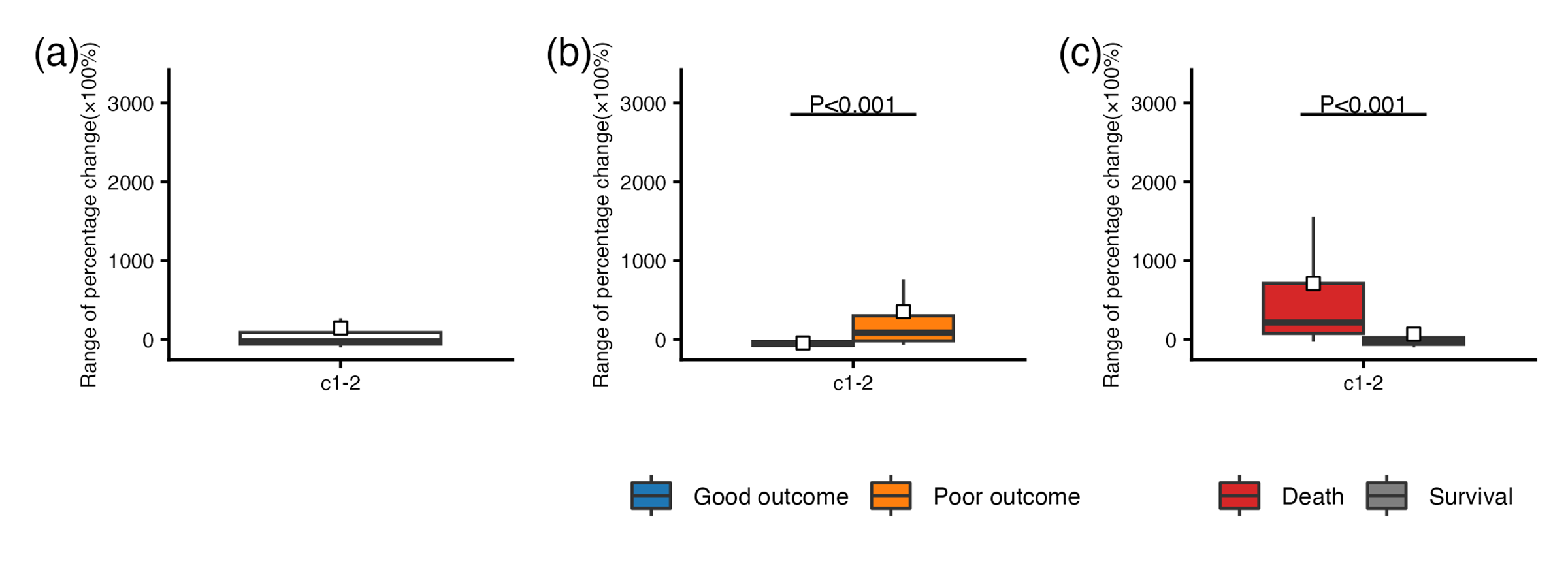
****Figure** S2.** Percentage changes in NLR from T1 to T2: (a) overall; (b) by 3-month functional outcome; (c) by 3-month survival. White squares = means, boxes = IQRs, central lines = medians. P values from Wilcoxon rank-sum test; c1–2 denotes T1→T2. Abbreviations: NLR，neutrophil-to-lymphocyte ratio; IQR, interquartile range; T1/T2 as in Figure 2.

**
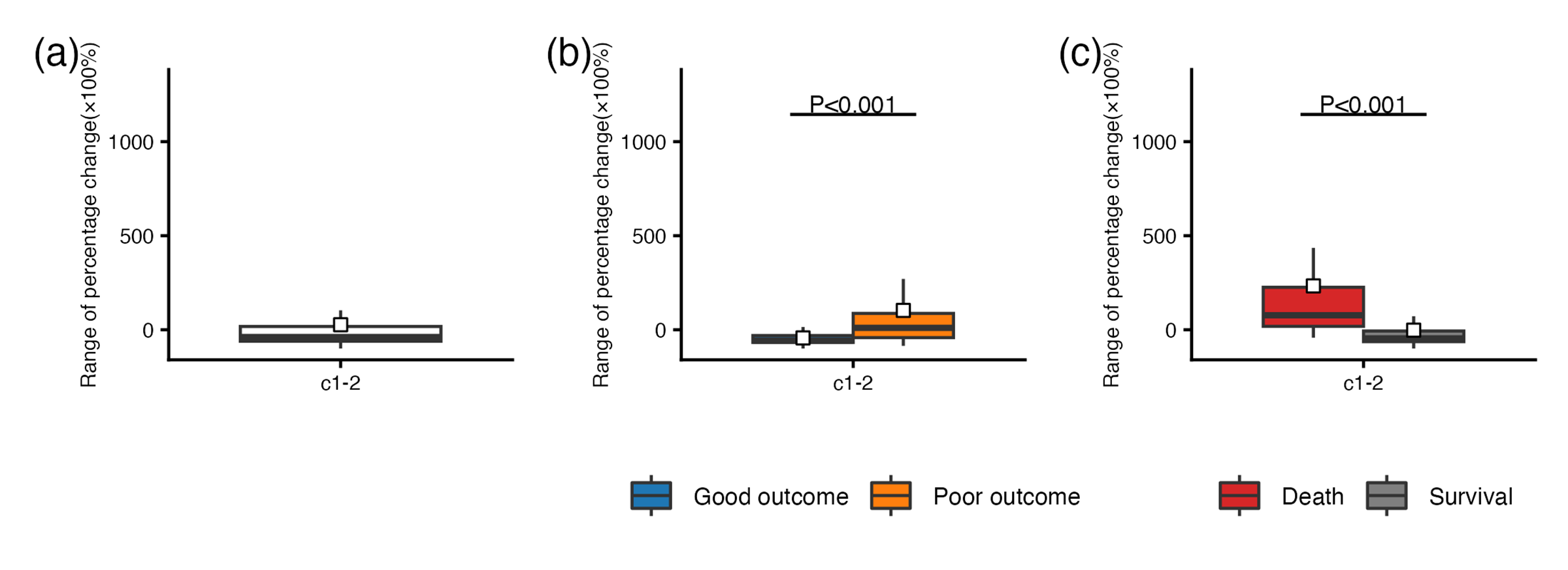
**Figure** S3.** Percentage changes in SII from T1 to T2: (a) overall; (b) by 3-month functional outcome; (c) by 3-month survival. White squares = means, boxes = IQRs, central lines = medians. P values from Wilcoxon rank-sum test; c1–2 denotes T1→T2. Poor outcome and death groups showed greater changes than controls.Abbreviations: SII, systemic immune-inflammation index; IQR, interquartile range; T1/T2 as in Figure 2.

**
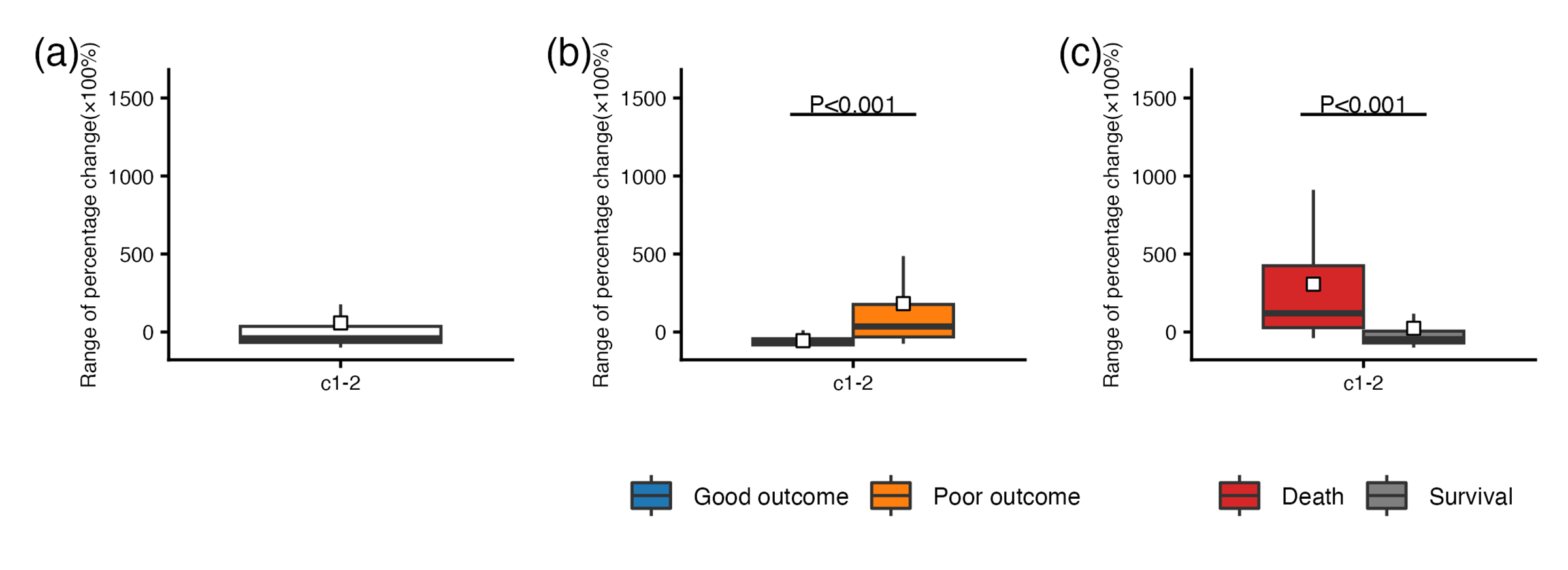
**

****Figure** S4.** Percentage changes in SIRI from T1 to T2: (a) overall; (b) by 3-month functional outcome; (c) by 3-month survival. White squares = means, boxes = IQRs, central lines = medians. P values from Wilcoxon rank-sum test; c1–2 denotes T1→T2. Abbreviations: SIRI, systemic inflammation response index; IQR, interquartile range; T1/T2 as in Figure 2.

**
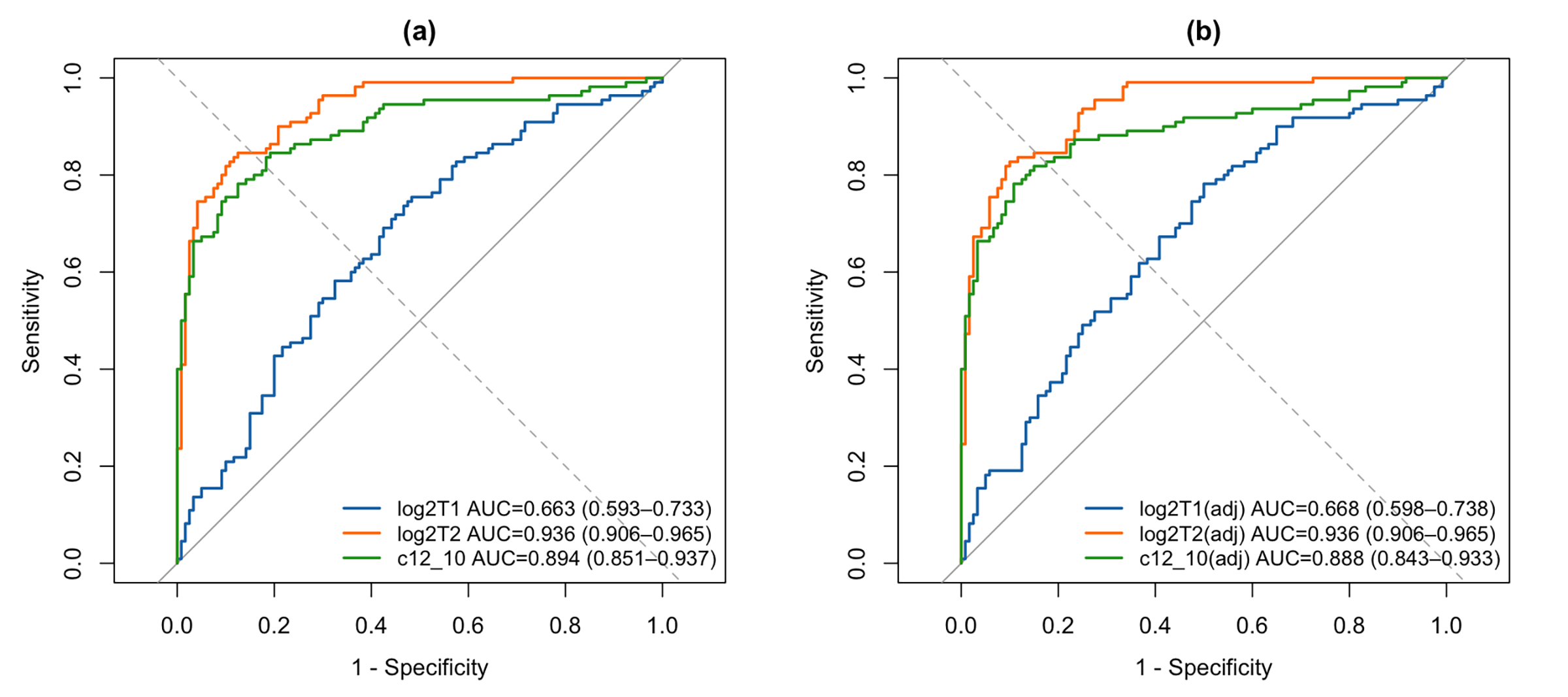
**Figure** S5.** ROC curves of IPI for 3-month poor outcome.**(a) Unadjusted model; (b) Adjusted model (age, sex). C**urves: logT1, logT2, c12; Legends show AUC with 95% CI. Abbreviations: ROC, receiver operating characteristic; AUC, area under the curve; CI, confidence interval; IPI, inflammation prognostic index

**
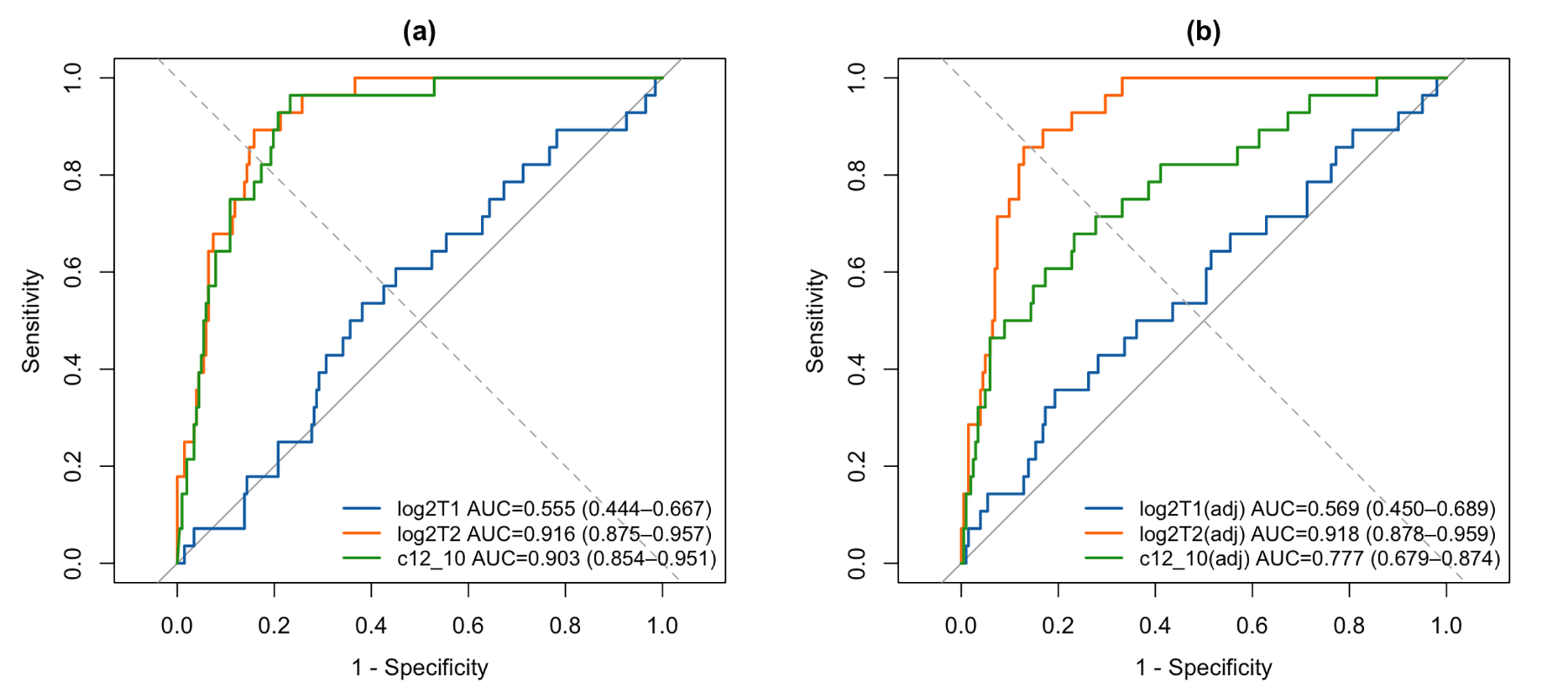
**Figure** S6.** ROC curves of IPI for mortality **(a) Unadjusted; (b) Adjusted.** Curves: logT1, logT2, c12; AUC with 95% CI. Abbreviations as in Figure 9.

**
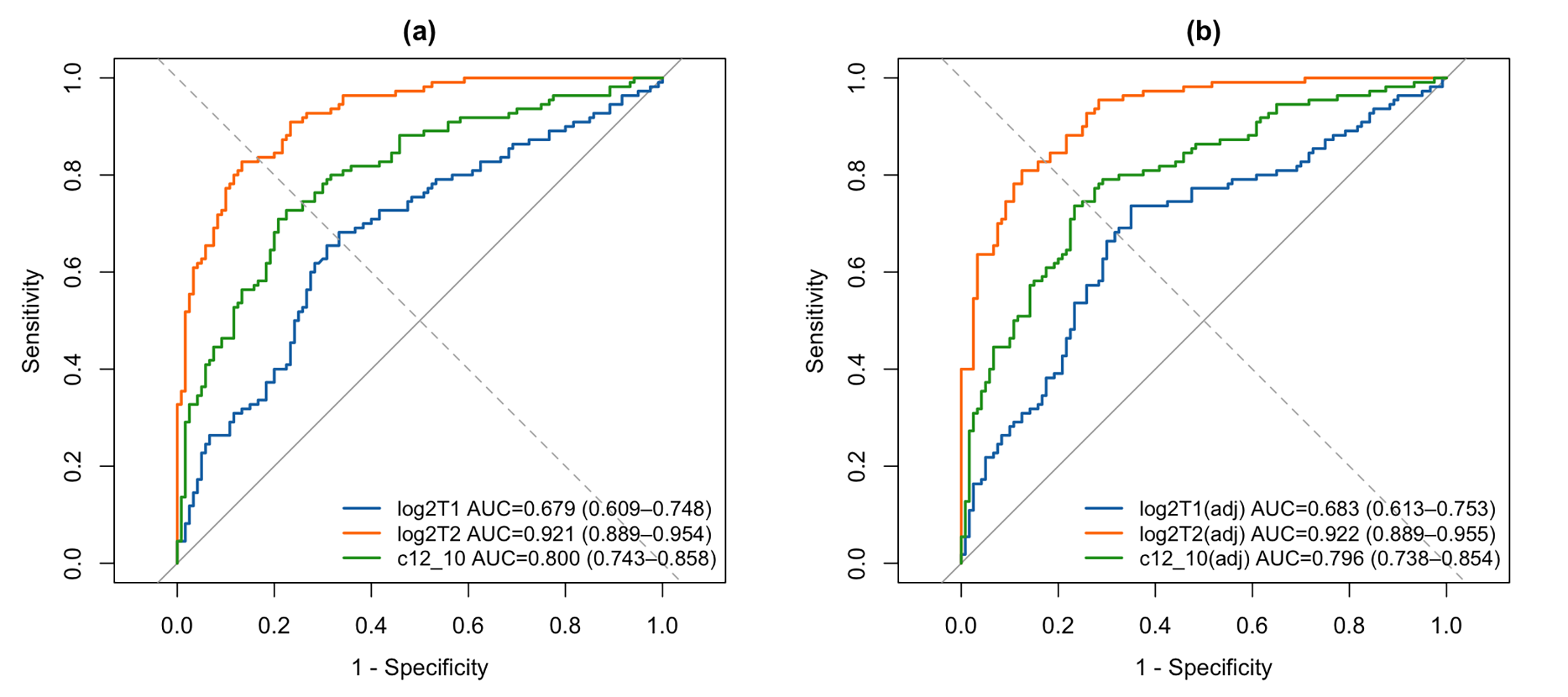
**Figure** S7.** ROC curves of NLR for 3-month poor outcome.**(a) Unadjusted; (b) Adjusted.** Curves: logT1, logT2, c12; AUC with 95% CI. Abbreviations: NLR，neutrophil-to-lymphocyte ratio; others as in Figure 9.

**
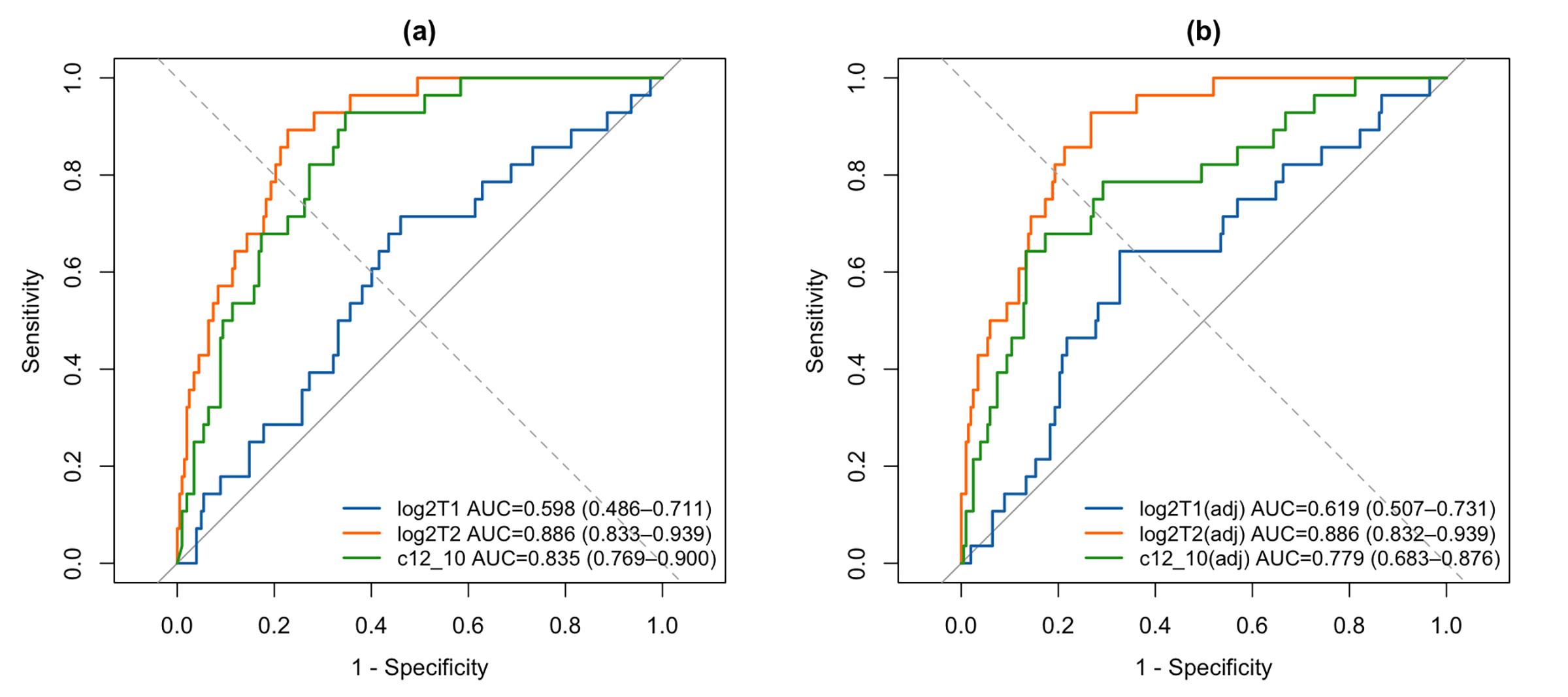
**Figure** S8.** ROC curves of NLR for mortality;**(a) Unadjusted; (b) Adjusted.** Curves: logT1, logT2, c12; AUC with 95% CI. Abbreviations as in Figure 11.

**
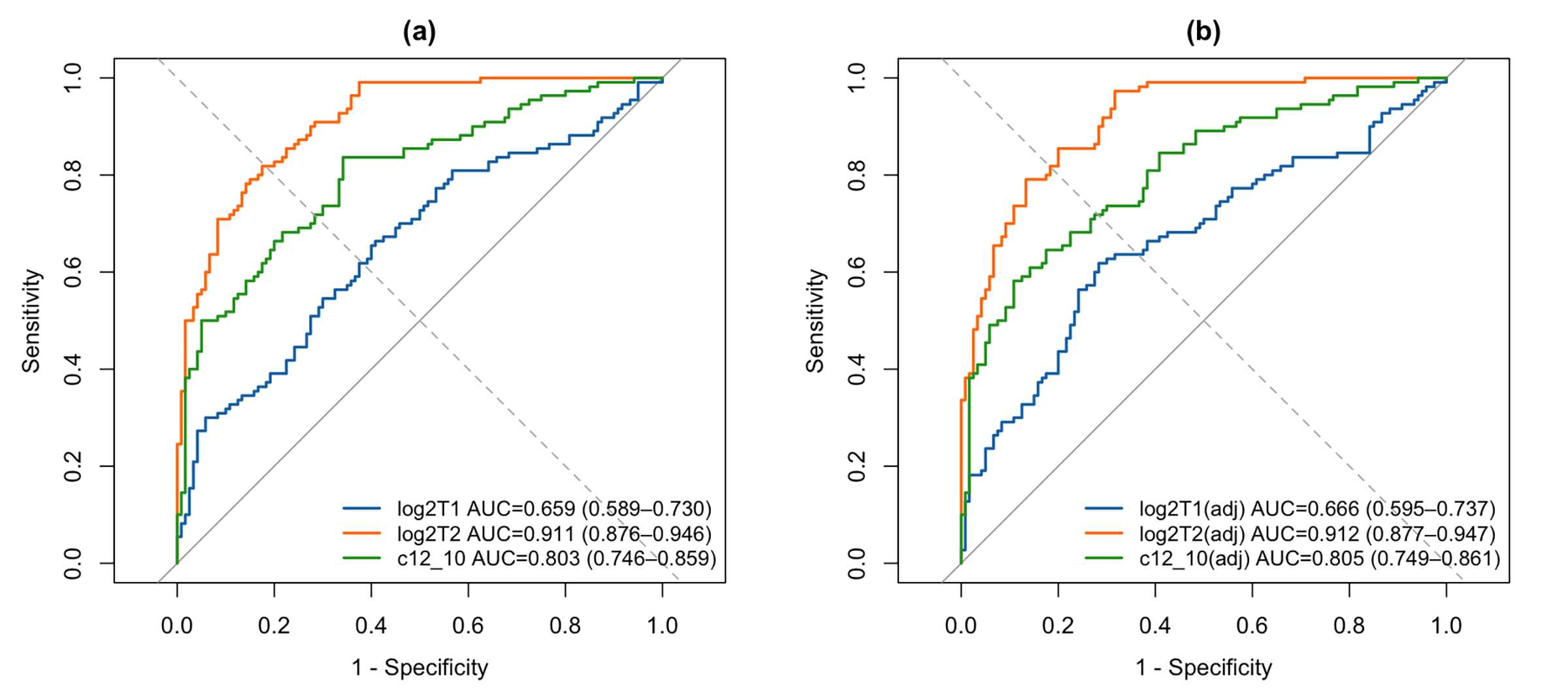
**Figure** S9.** ROC curves of SII for 3-month poor outcome; **(a) Unadjusted; (b) Adjusted.** Curves: logT1, logT2, c12; AUC with 95% CI. Abbreviations: SII, systemic immune-inflammation index; others as in Figure 9.

**
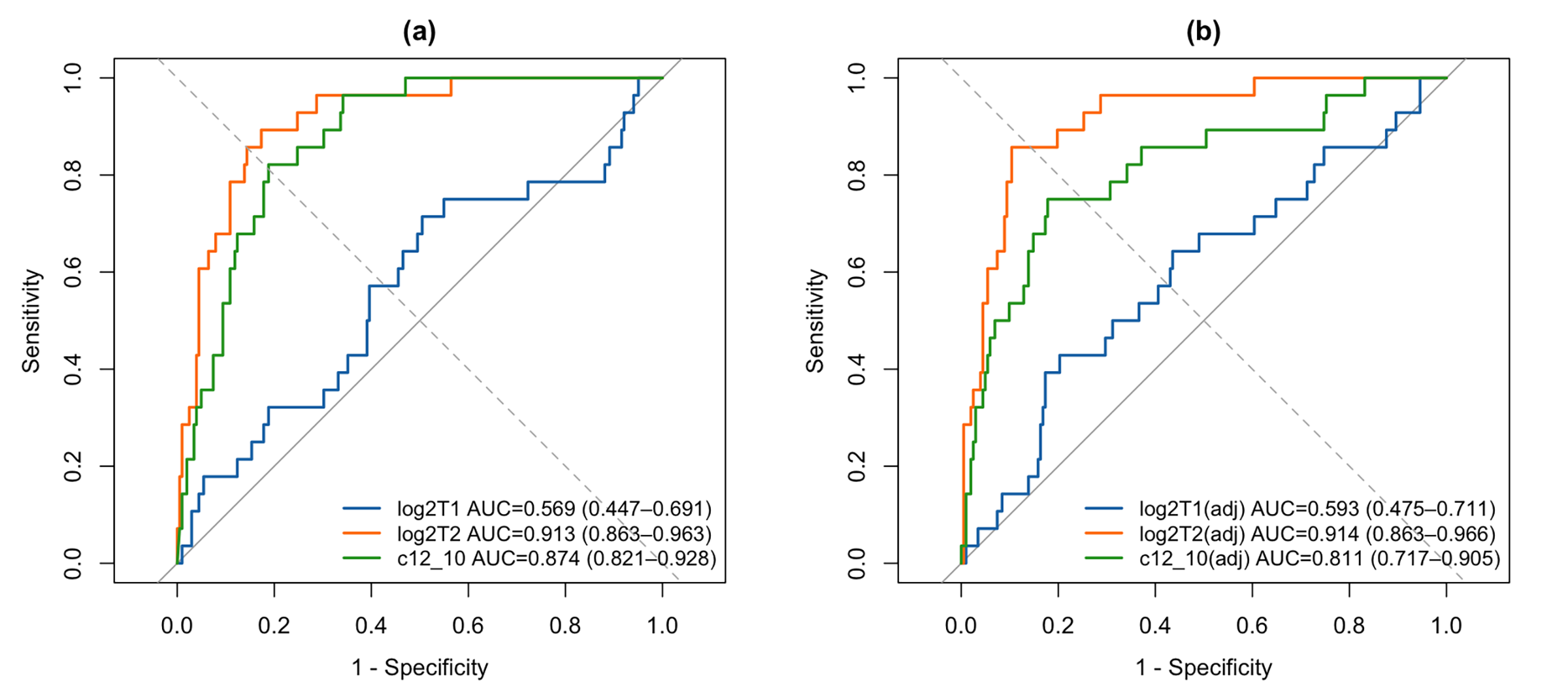
**Figure** S10.** ROC curves of SII for mortality ; **(a) Unadjusted; (b) Adjusted.** Curves: logT1, logT2, c12; AUC with 95% CI. Abbreviations as in Figure 13.

**
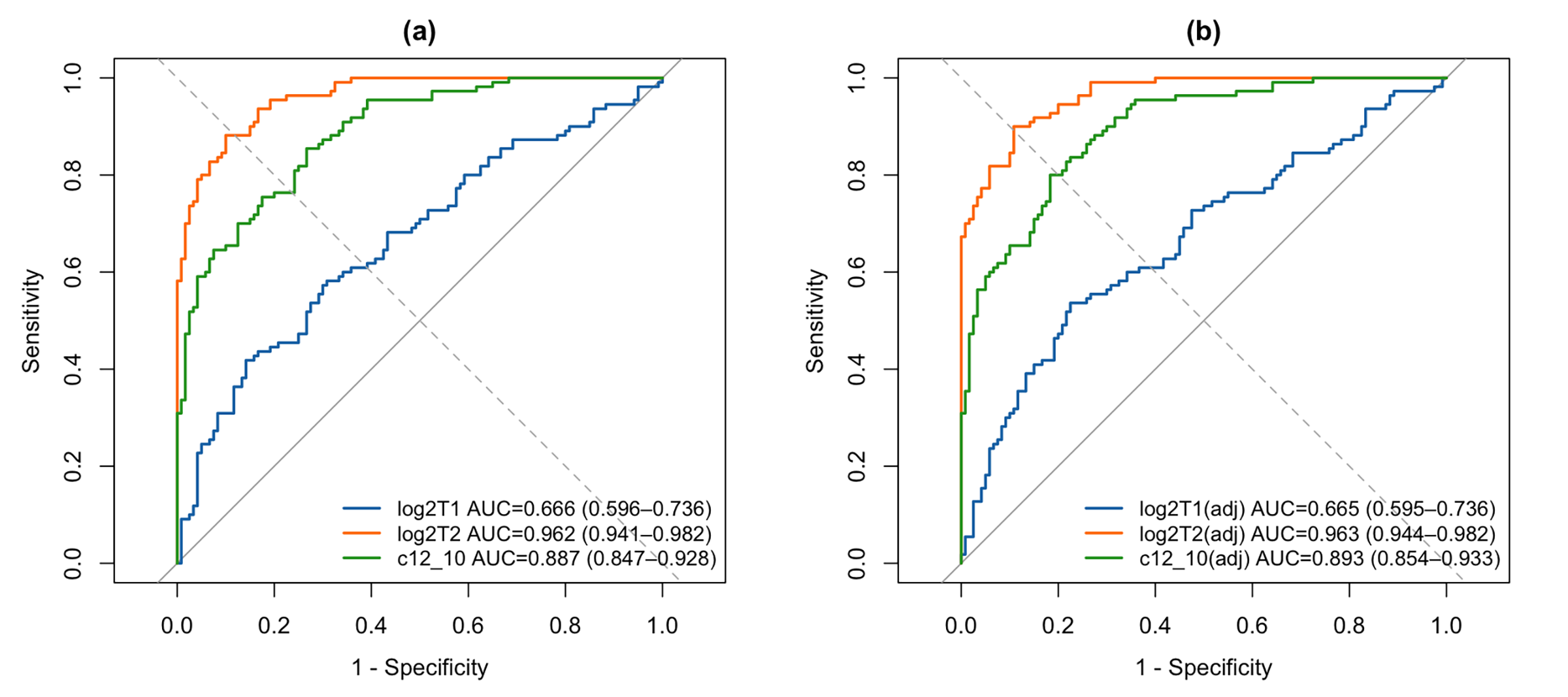
**Figure** S11.** ROC curves of SIRI for 3-month poor outcome; **(a) Unadjusted; (b) Adjusted.** Curves: logT1, logT2, c12; AUC with 95% CI. Abbreviations: SIRI, systemic inflammation response index; others as in Figure 9.

****
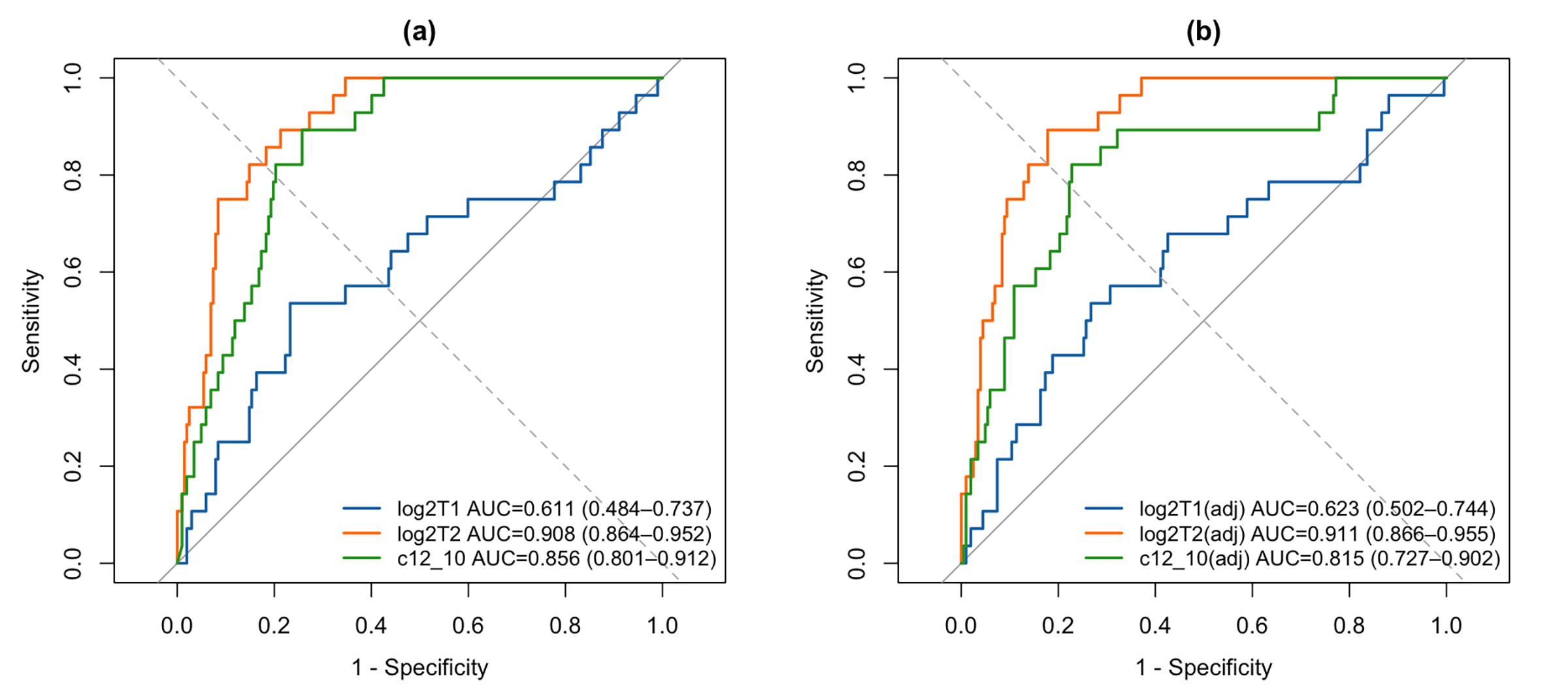
Figure** S12**.**** ROC curves of SIRI for mortality ; **(a) Unadjusted; (b) Adjusted.** Curves: logT1, logT2, c12; AUC with 95% CI. Abbreviations as in Figure 15.
